# Increasing referrals to emergency department for psychiatric consultation and treatment among 50,056 adolescents and young adults: Predictors and implications

**DOI:** 10.1111/jcv2.12016

**Published:** 2021-06-26

**Authors:** Marco Solmi, Foscarina Della Rocca, Umberto Granziol, Angela Favaro, Miranda Zoleo, Carla Cremonese

**Affiliations:** ^1^ Neurosciences Department University of Padua Padua Italy; ^2^ Padua Neuroscience Center University of Padua Padua Italy; ^3^ Psychiatry Unit Padua University Hospital Padua Italy; ^4^ Emergency Department Padua University Hospital Padua Italy; ^5^ Department of General Psychology University of Padua Padova Italy

**Keywords:** adolescent psychiatry, early intervention, emergency, epidemiology, prevention, psychiatry in Europe

## Abstract

**Objectives:**

To identify predictors and time trends over 10 years of psychiatric consultation or treatment in adolescents and young adults referring to Emergency Department (ED).

**Methods:**

Real‐world cohort data from 50,056 adolescents and young adults referring 105,596 times to ED between 2007 and 2016. We tested whether gender, age, triage code (red, yellow, green, white with decreasing severity), and referral modality predicted primary (psychiatric consultation) or secondary outcomes (anxiolytic treatment, sedative treatment, psychiatric admission), and whether these outcomes increased over the last 10 years.

**Results:**

Mean age was 19.57(SD = 2.52), female percentage was 48.77%. Overall, 6.93% underwent psychiatric consultation, treatment, or admission. Among 2,547 adolescents and young adults undergoing a psychiatric consultation, 58.07% had either yellow or red triage code, and 47.2% were brought by ambulance. Female gender predicted psychiatric consultation and anxiolytic treatment, male gender predicted sedative treatment, suggesting gender differences in help‐seeking behaviors. Older age predicted all outcomes. Severe triage presentation and being brought by ambulance increased the risk of primary and secondary outcomes. Psychiatric consultation (1.77% to 3.64%), anxiolytic (3.04% to 6.15%), or admission (0.40% to 0.98%) roughly doubled, and sedative (0.27% to 1.23%) treatment had a four‐fold increase from 2007 to 2016.

**Conclusions:**

Among adolescents and young adults aged 15 to 24 years old ED appears to be necessary for young help‐seeking subjects given the severe presentations and the increasing number of adolescents referring to ED. More studies should assess whether ED might be helpful in detecting subjects with sub‐threshold or early psychiatric symptoms, or at risk for severe mental illness.

## Graphical abstract

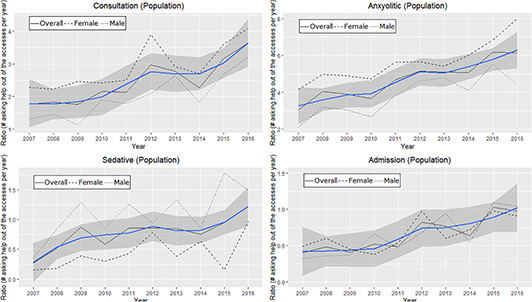

## INTRODUCTION

Patients with severe mental illness (SMI) have a decreased life expectancy compared with general population of around 15 to 20 years (Hjorthøj et al., 2017; Laursen et al., 2012). Natural causes (not suicide) account for more than 70% of lost life‐years in patients with SMI (Jayatilleke et al., 2017). Among medical comorbidities, compelling evidence supports an association between severe mental illness and diabetes (Vancampfort et al., 2016), metabolic syndrome (Vancampfort et al., 2015), and cardiovascular disease (Correll et al., 2017). Given the frequent medical comorbidity patients with SMI frequently access to Emergency Department (ED) for medical reasons. In addition to medical emergencies or car crash (Gianfranchi et al., 2017), patients can frequently refer to ED for psychiatric symptoms when relapses occur. ED might be relevant for young populations in particular, given that the median age of onset of any mental disorder is around 18 years old (Solmi et al., 2021) (18 for eating disorders [Solmi et al., 2021], 17 for anxiety disorders [Solmi et al., 2021], 19 for obsessive‐compulsive disorder [Solmi et al., 2021], 25 for schizophrenia and related disorders [Solmi et al., 2021], 25 for substance‐related disorders [Solmi et al., 2021; Yin et al., 2018], 31 for depressive or bipolar disorders [Caspi et al., 2020; Kessler et al., 2005; Solmi et al., 2021; Vaingankar et al., 2013]). Importantly, beyond accessing to ED for physical symptoms, or for symptoms of full blown mental disorders, patients often present psychiatric subthreshold symptoms well before SMI onset, often between age 15 and 24 years old (Kessler et al., 2007). Concerning evidence from the United States has shown increasing rates of adolescents accessing to ED, with suicidal ideation or because of suicide attempt (Burstein et al., 2019). Also, reports from Italy have shown that psychiatric consultations, suicidal behavior, suicidal ideation, and non‐suicidal self‐injury have increased from 7.7% in 2011 to 19% in 2016, in particular in females (Castaldo et al., 2020). Hence, according to this body of evidence, it could be expected that ED may serve as an early detection opportunity, and that frequently adolescents or young adults presenting to ED can have severe clinical pictures. Evidence showing that adolescents accessing to ED have severe psychiatric clinical presentations is of upmost clinical relevance, since it defines the necessary role of ED in the pathway to care and clinical services within a wider heterogeneous setting of separate yet integrated services collaborating for prevention/early intervention for psychiatric disorders in adolescents/young adults.

**Key Points**

- Referrals of adolescents to Emergency Department (ED) have been increasing over the last 10 years. The request for psychiatric consultation had a 2.05‐fold increase from 2007 (1.77%) to 2016 (3.64%), with an average year‐to‐year growth rate of 9.99%
- Over the last 10 years, the percentage of adolescents accessing to ED and requiring anxiolytic treatment, or admission in psychiatry unit had about a 2‐fold increase. The adolescent requiring a sedative treatment had a 4.5‐fold increase
- Presentation and need for treatment differ between males and females
- Adolescents accessing to ED in need of psychiatric care have severe presentations and triage codes
- EDs should be involved in the network of services for prevention and early intervention for mental disorders

However, service organization and pathway to care is different across countries, and within regions in the same country. For instance, findings from insurance‐based health system cannot be generalized to public funded health systems. Similarly, reports from rural areas might differ substantially from center with high urbanicity. Hence, in order to inform local service organization, local evidence must be generated. Specifically, to inform whether ED can have a role in early detection of adolescents and young adults with symptoms of psychiatric disorders, predictors, rates, and time trends of indicators of psychiatric symptoms, as well as severity of clinical presentation must be identified using data from local context.

The main aim of the present work is to describe which ED referral (i.e., severity of presentation) and subject characteristics (i.e., demographics) predict psychiatric consultation, treatment, or admission (as a proxy of psychiatric symptoms), and to test whether ED referrals due to psychiatric symptoms in young subjects have increased over the last 10 years. Also, we aim to show that specialist psychiatric assessment in ED is sub‐optimally delivered. Our hypotheses are that ED is a necessary setting for a safe evaluation of help‐seeking subjects referring to ED with psychiatric symptoms (in other words, that ED could not be replaced by different setting with lower medical assistance), that adolescents and young adults frequently access, and that referrals to ED for psychiatric symptoms have increased over the last 10 years.

## METHODS

### Population, study design, variables of interest, primary and secondary outcomes

These real‐world data were extracted from clinical electronic records from a retrospective cohort study, approved by local Ethical Committee. It included all subjects aged between 15 (adolescents access to ED since age 15) and 24 years old (upper limit widely used for young adults) who accessed for any reason to Padua University Hospital ED between 2007 and 2016. Regarding the hospital setting, Padua University Hospital ED provides emergency care to around 250,000 citizens, plus a population of around 60,000 students attending University of Padua courses. Even though there is an additional ED in the same area (Sant. Antonio Hospital), all psychiatric consultations and care are centralized in Padua University Hospital ED. Predictors were year of access to ED, age, triage code (ranging from white, lowest severity to red, highest severity with potential life‐threatening course), referral modality (ambulance with or without doctor, autonomously, other), and gender. More in detail, regarding triage code, if vital functions are compromised the patient receives a red color code and has the highest treatment priority (e.g., cardiac arrest, respiratory distress, and coma). When clinical condition of the patient is stable but symptoms indicate a risk of rapid deterioration she/he receives a yellow color code (e.g., syncope, chest pain, and palpitations); otherwise the patient has no risk priority ad receives a green or white color code on the basis of degree of pain. The assignment of each specific triage code implies an operational response: red‐coded patients have immediate access to the treatment areas, yellow‐coded patient have access to protected waiting areas where they can wait for medical evaluation at most 20 min. The patients without risk priority can wait in a common waiting room without time limits. Primary outcomes was psychiatric consultation. Secondary outcomes were anxiolytic drug administration (diazepam), sedative drugs administration (haloperidol, promazine, or midazolam), and psychiatric admission.

We estimated which characteristics predicted primary and secondary outcomes, and whether primary and secondary outcomes increased over time. We also calculated rates of subjects not undergoing a psychiatric consultation despite being treated with anxiolytic treatment, as a proxy of sub‐optimal psychiatric specialist evaluation delivery.

### Statistical analyses

We tested multivariate generalized linear mixed effect models (GLMMs). In the analyses to individuate factors associated with psychiatric consultation and treatment, fixed factors of the multivariate models (and preceding primary and secondary outcomes) were gender, age at the time of arrival in ED, triage code (four levels: white, green, yellow, and red), and the referral modality (four levels: ambulance without medical doctor, ambulance with medical doctor, autonomous, and other ways). Considering that some people underwent into ED multiple times, we set the individual tax code as the clustering variable and the intercept as random factor (i.e., random intercept models). We used time series analysis to investigate the trend of psychiatric consultation, treatment, and admission over 10 years. In particular, we calculated the year‐over‐year change in ratio between outcomes and total ED accesses from 2007 to 2016, and we tested if this change had an increasing trend. We did not consider time to event and did not run a survival analysis due to the short time between exposure (referral to ED) and outcome (psychiatric consultation in ED). We computed all the statistical analysis by means of the R statistical software (R Foundation for Statistical Computing, 2019). We used the *lme4* package (Bates et al., 2015) to test the GLMMs, *tfplot* (Gilbert, 2015) and *pastecs* (Grosjean & Ibanez, 2018) packages to calculate the year‐over‐year change and the trend statistics, respectively. Finally, we estimated the *R* ^2^ of the GLMMs by means of the *MuMin* package (Barton, 2013). More specifically, we calculated both marginal and conditional *R* (Laursen et al., 2012), referring, respectively, to the proportion of variance explained by only the fixed factors and by both the fixed and random factors (Nakagawa & Schielzeth, 2013). Analyses scripts are provided in Appendix S1.

## RESULTS

### Population

We included 50,056 subjects aged between 15 and 24, who accessed to Padua University Hospital ED 105,596 times. Mean age was 19.57(SD = 2.52), 48.77% were females. Overall patients were assigned with a white triage code in 67.07%, green in 9.46%, yellow in 21.75%, and red in 1.73%. Referral modality was autonomously in 73.80%, in other ways 6.51%, brought by ambulance without medical doctor in 17.52%, and by ambulance with medical doctor in 2.16%. Among the whole sample, 2.41% of subjects aged 15 to 24 years old referring to ED underwent psychiatric consultation, 4.66% underwent anxiolytic treatment, 0.77% underwent sedative treatment, and 0.64% underwent psychiatric admission. Overall, 6.93% of young subjects referring to ED needed at least one among psychiatric consultation, anxiolytic treatment, sedative treatment, or psychiatric admission. Also, among 2,547 subjects undergoing a psychiatric consultation, 30.43% subjects had white triage code, 11.5% green code, 52.53% yellow code, 5.54% red code, and 42.21X% were brought by ambulance without doctor, 4.99% by ambulance with doctor, 45.74% autonomously, and 7.07% in other ways.

### Predictors of primary outcome

Compared to female, male subjects were less likely to undergo a psychiatric consultation (OR = 0.65, 95%CI 0.45–0.95, *p* = 0.23) (Table 1). Moreover, older age predicted a psychiatric consultation (OR = 1.06, 95%CI 1.01–1.11, *p* = 0.008), as green (OR = 2.85, 95%CI 2.17–3.75, *p* < 0.001), yellow (OR 6.99, 95%CI 5.71–8.56, *p* < 0.001), and red triage codes (OR = 7.45, 95%CI 4.92–11.27, *p* < 0.001) did over whit code. Considering referral modality, compared with autonomously referring to ED, being brought by other modalities other than ambulance (i.e., by family members) (OR = 1.92, 95%CI 1.38–2.68, *p* < 0.001), by ambulance but without medical doctor (OR = 2.75, 2.26–3.34, *p* < 0.001), or by ambulance with doctor (OR = 2.48, 95%CI 1.62–3.80, *p* < 0.001) were associated with psychiatric consultation. Overall, the model explained 98.13% of the variance.

**TABLE 1:** Emergency Department referral characteristics and psychiatric consultation in 50,056 subjects aged 15 to 25 years old

Characteristics of referral to Emergency Department		Psychiatric consultation %	OR (95%CI)	*p*‐value
	Female	2.88%	Reference	
	Male	1.96%	0.65 (0.451–0.951)	0.023
	Age	‐	1.06 (1.015–1.107)	0.008
Triage	Triage white	1.09%	Reference	
	Triage green	2.94%	2.85 (2.173–3.752)	<0.001
	Triage yellow	5.83%	6.99 (5.711–8.555)	<0.001
	Triage red	7.74%	7.45 (4.923–11.274)	<0.001
Referral modality	Autonomy	1.49%	Reference	
	Other	2.88%	1.92 (1.382–2.680)	<0.001
	Ambulance without doctor	5.81%	2.75 (2.263–3.341)	<0.001
	Ambulance with doctor	5.95%	2.48 (1.619–3.805)	<0.001
Marginal *R* ^2^	0.68%			
Conditional *R* ^2^	98.13%			

### Predictors of secondary outcomes

#### Anxiolytic treatment

Compared to female, male subjects were less likely to undergo anxiolytic treatment (OR = 0.61, 95%CI 0.48–‐0.77, *p* < 0.001) (Table 2). Older age predicted anxiolytic treatment (OR = 1.2, 95%CI 1.16–1.24, *p* < 0.001), as green (OR = 6.13, 95%CI 5.14–7.32, *p* < 0.001), yellow (OR = 6.63, 95%CI 5.75–7.65, *p* < 0.001), and red triage codes (OR = 16.29, 95%CI 11.86–22.40 *p* < 0.001) did over white triage code. As regards referral modality, compared with autonomously referring to ED, being brought by other modalities (OR = 1.56, 95%CI 1.24–1.96, *p* < 0.001) or by ambulance without medical doctor (OR 2.08, 95%CI 1.80–2.40, *p* < 0.001) was associated with anxiolytic treatment, while being brought by ambulance with medical doctor did not make any difference. Overall, the model explained 97.5% of the variance.

**TABLE 2:** Emergency Department referral characteristics and anxiolytic treatment in 50,056 subjects aged 15 to 25 years old

Characteristics of referral to Emergency Department	Anxiolytic treatment %	OR (95%CI)	*p*‐value
Female	5.59%	Reference	
Male	3.76%	0.61 (0.483–0.776)	<0.001
Age	‐	1.2 (1.164–1.239)	<0.001
Triage			
Triage white	2.20%	Reference	
Triage green	8.06%	6.13 (5.142–7.321)	<0.001
Triage yellow	9.81%	6.63 (5.747–7.649)	<0.001
Triage red	14.11%	16.29 (11.858–22.399)	<0.001
Referral modality			
Autonomy	3.66%	Reference	
Other	5.72%	1.56 (1.243–1.955)	<0.01
Ambulance with doctor	5.86%	0.81 (0.562–1.161)	0.24
Ambulance without doctor	8.49%	2.08 (1.805–2.404)	<0.001
Marginal *R* ^2^	10.07%		
Conditional *R* ^2^	97.5%		

#### Sedative treatment

Factors associated with sedative treatment are reported in Table 3. Male (OR = 1.41, *p* < 0.001) and older subjects (OR = 1.01, 95%CI 1.01–1.01, *p* < 0.001) predicted treatment with a sedative (Table 3), as did green (OR = 3.7, 95%CI 2.16–6.34, *p* < 0.001), yellow (OR = 9.75, 95%CI 7.06–13.46, *p* < 0.001), and red codes (OR = 115.88, 95%CI 115.82–115.95, *p* < 0.001) over white triage code. As regards referral modality, compared with autonomously referring to ED, being brought by ambulance without medical doctor (OR = 3.6, 95%CI 2.61–4.97, *p* < 0.001), by ambulance with medical doctor (OR = 23.15, 95%CI 23.14–23.16, *p* < 0.001), or being brought other modalities other than ambulance (i.e., by family members; OR = 2.69, 95%CI 1.36–5.34, *p* < 0.001) was associated with sedative treatment. Overall, the model explained 97.5% of the variance.

**TABLE 3:** Emergency Department referral characteristics and sedative treatment in 50,056 subjects aged 15 to 25 years old

Characteristics of referral to Emergency Department	Sedative treatment	OR (95%CI)	*p*‐value
Female	0.43%	Reference	
Male	1.08%	1.41 (1.414–1.416)	<0.001
Age	‐	1.01 (1.012–1.013)	<0.001
Triage			
Triage white	0.09%	Reference	
Triage green	0.56%	3.7 (2.162–6.339)	<0.001
Triage yellow	1.67%	9.75 (7.064–13.46)	<0.001
Triage red	16.68%	115.88 (115.822–115.95)	<0.001
Referral modality			
Autonomy	0.19%	Reference	
Other	0.47%	2.69 (1.358–5.339)	<0.01
Ambulance without doctor	1.53%	3.6 (2.607–4.975)	<0.001
Ambulance with doctor	14.33%	23.15 (23.137–23.163)	<0.001
Marginal *R* ^2^	17.23%		
Conditional *R* ^2^	97.5%		

#### Psychiatric admission

Psychiatric admission was independent from gender (Table 4). Older age predicted psychiatric inpatients admission (OR = 1.3, 95%CI 1.19–1.43, *p* < 0.001), as did green (OR = 4.01, 95%CI 2.44–6.60, *p* < 0.001), yellow (OR = 9.92, 95%CI 6.62–14.87, *p* < 0.001), and red codes (OR = 12.21, 95%CI 8.31–17.94, *p* < 0.001) over white triage code. As regards referral modality, compared with autonomously referring to ED, being brought by ambulance without medical doctor (OR = 1.79, 95%CI 1.29–2.51, *p* < 0.001), or by ambulance with medical doctor (OR = 6.47, 95%CI 321–13.05, *p* < 0.001) was associated with psychiatric admission, while being brought by other modalities other than ambulance (i.e., by family members) did not make any difference. Overall, the model explained 98.04% of the variance.

**TABLE 4:** Emergency Department referral characteristics and psychiatric admission in 50,056 subjects aged 15 to 25 years old

Characteristics of referral to Emergency Department	Psychiatric admission %	OR (95%CI)	*p*‐value
Female	0.63%	Reference	
Male	0.66%	0.82 (0.400–1.680)	0.58
Age	‐	1.3 (1.191–1.435)	<0.001
Triage			
Triage white	0.17%	Reference	
Triage green	0.73%	4.01 (2.442–6.599)	<0.001
Triage yellow	1.95%	9.92 (6.619–14.875)	<0.001
Triage red	1.64%	12.21 (8.310–17.941)	<0.001
Referral modality			
Autonomy	0.34%	Reference	
Other	0.66%	1.3 (0.680–2.498)	0.42
Ambulance without doctor	1.68%	1.79 (1.287–2.511)	<0.001
Ambulance with doctor	2.62%	6.47 (3.212–13.046)	<0.001
Marginal *R* ^2^	0.95%		
Conditional *R* ^2^	98.04%		

## TRENDS OF PSYCHIATRIC CONSULTATION AND TREATMENT OVER TIME

**FIGURE 1:**
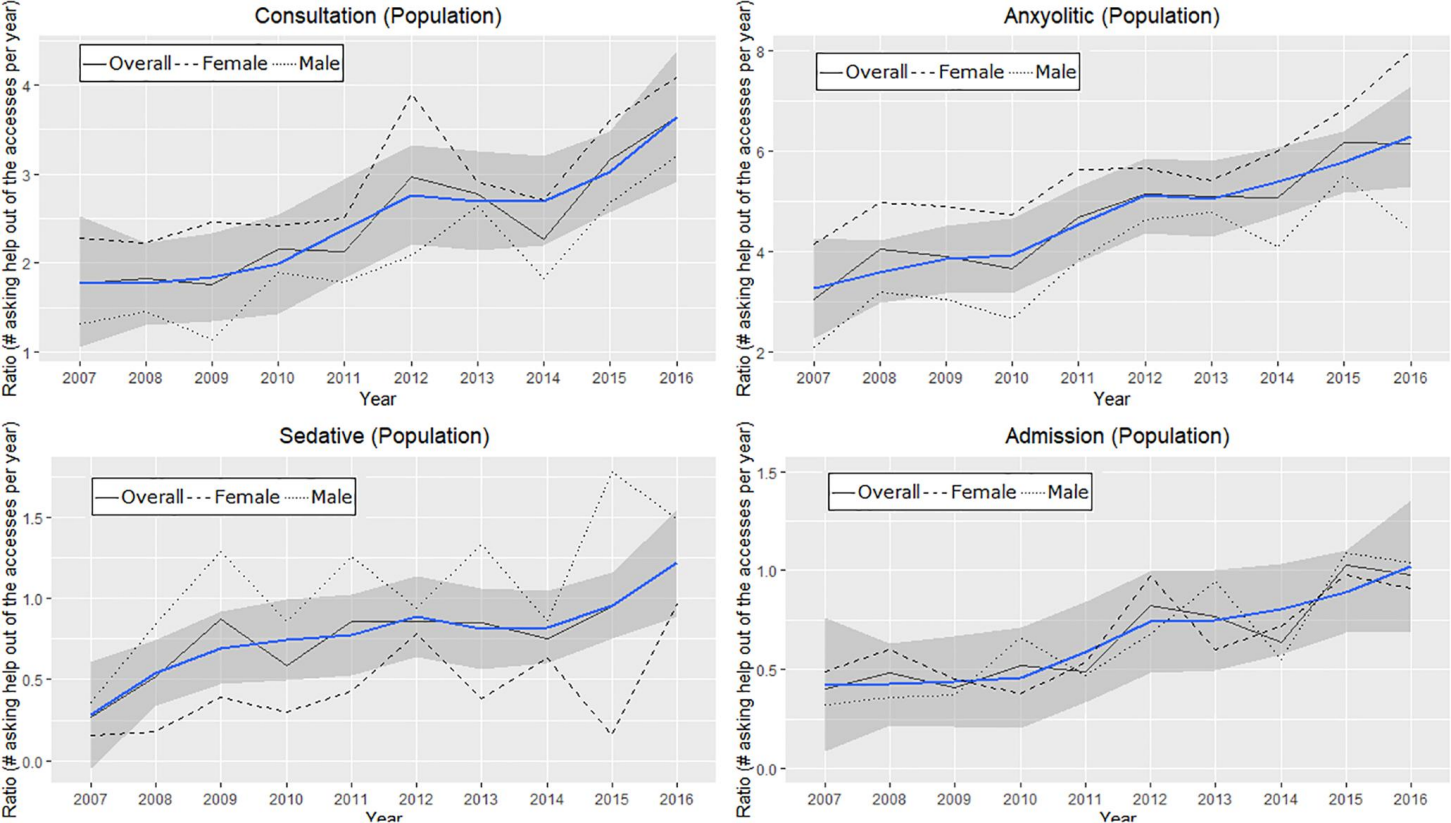
Emergency Department psychiatric consultation and treatment time trends in over 10 years in 50,056 subjects aged 15 to 25 years old. Black line connects proportion rates over years, blue line is the fitted line, and the shaded area indicate the 95% confidence interval. Continues lines represent all sample, dotted line represents males, and dashed line represents females

Rates of psychiatric consultations (*ρ* = 0.9, *p* < 0.001; mean percentage of increase = 9.99%), anxiolytic treatment (*ρ* = 0.89, *p* < 0.01, mean percentage of increase = 9.02%), sedative treatment (*ρ* = 0.7, *p* = 0.02; mean percentage of increase = 24.08%), and psychiatric admission (*ρ* = 0.91, *p* < 0.001, *p* < 0.005; mean percentage of increase = 14.1%) increased from 2007 to 2017 (Table 5, Figure 1).

**TABLE 5:** Emergency Department psychiatric consultation and treatment frequencies over time in 50,056 subjects aged 15 to 25 years old

Year	Psychiatric consultation	Year‐to‐year percentage growth rate	Anxiolytic treatment	Year‐to‐year percentage growth rate	Sedative treatment	Year‐to‐year percentage growth rate	Psychiatric admission	Year‐to‐year percentage growth rate
2007	1.77%		3.04%		0.27%		0.40%	
2008	1.82%	2.82%	4.05%	33.22%	0.52%	92.59%	0.48%	20%
2009	1.76%	−3.29%	3.91%	−3.46%	0.87%	67.31%	0.41%	−14.58%
2010	2.16%	22.73%	3.66%	−6.39%	0.59%	−32.18%	0.52%	26.83%
2011	2.13%	−1.39%	4.69%	28.14%	0.86%	45.76%	0.49%	−5.77%
2012	2.97%	39.44%	5.14%	9.59%	0.86%	0%	0.82%	67.35%
2013	2.77%	−6.73%	5.10%	−0.78%	0.85%	−1.16%	0.77%	−6.1%
2014	2.27%	−18.05%	5.06%	−0.78%	0.75%	−11.76%	0.64%	−16.88%
2015	3.16%	39.21%	6.18%	22.13%	0.95%	26.67%	1.03%	60.94%
2016	3.64%	15.19%	6.15%	−0.48%	1.23%	29.47%	0.98%	−4.85%
Hypothesis testing for ascending trend	*ρ* = 0.9 *p* < 0.001		*ρ* = 0.89 *p* < 0.01		*ρ* = 0.7 *p* = 0.02		*ρ* = 0.91 *p* < 0.001	
Ascending trend for females	*ρ* = 0.89 *p* < 0.01		*ρ* = 0.91 *p* < 0.001		*ρ* = 0.5 *p* = 0.14		*ρ* = 0.73 *p* = 0.02	
Ascending trend for males	*ρ* = 0.87 *p* < 0.01		*ρ* = 0.82 *p* < 0.01		*ρ* = 0.74 *p* = 0.02		*ρ* = 0.89 *p* < 0.01	

Significantly increased trends have been found in males and females separately.

## SUBOPTIMAL PSYCHIATRIC CONSULTATION DELIVERY IN ED

We confirm that mental status evaluation is sub‐optimally delivered in ED since a psychiatric consultation is requested only in a portion of patients (2.41%) who are treated with anxiolytic treatment (4.66%).

## DISCUSSION

Real‐world data from 50,056 subjects aged between 15 and 24 years old referring 105,596 time to ED between 2007 and 2016 show that adolescents undergoing psychiatric consultation often have severe presentations. Moreover, we show that ED referrals occur following two different patterns based on gender, with females showing more help‐seeking behavior and so undergoing more frequently a psychiatric consultation or anxiolytic treatment, while males presenting to ED with more severe clinical pictures probably associated with acute behavioral manifestations. Moreover, an increasing number of adolescents and young adults have been referring to ED for psychiatric symptoms over 10 years between 2007 and 2016. Finally, we confirm that there is the need to improve mental status assessment in young subjects referring to ED.

These results show that ED is a proper setting to assess mental status of adolescents with psychiatric symptoms with acute presentations. The small percentage of subjects accessing to ED and requiring psychiatric specialist assessment, do have severe presentations frequently coded as yellow or red code according to universal triage parameters (58.07%), and brought to ED by ambulance in almost one out of two cases (47.2%). Such cases could not have been safely and effectively examined and treated in clinical settings other than ED (i.e., psychiatric outpatients service, or by direct admissions to psychiatric inpatients unit without priori general medical assessment). Evaluating patients with SMI in ED should always prioritize the exclusion of medical conditions underlying behavioral or mental illness, and avoid the risk of underdiagnosing and undertreating medical comorbidities, which frequently occur in SMI (Al‐Seddik et al., 2019), largely more frequently than in general population (Correll et al., 2017). Such a risk of disparities in quality of medical care has already been shown, with patients suffering severe mental illness having lower rates of thrombolysis when affected by stroke compared with who does not have comorbid SMI (Bongiorno et al., 2019), for instance.

Beyond safety considerations supporting the role of ED in early mental assessment, we also describe gender‐differences between help‐seeking behaviors in ED. Among other factors, stigma could be one of the reasons why males show less help‐seeking behavior, given the association between male gender and stigma, and between stigma and less help‐seeking behavior (Clement et al., 2015; Zaninotto et al., 2018). However, reports from other settings actually describe that male gender is associated with higher ED access for mental disorders (Young et al., 2005). Beyond what might obstacle help‐seeking behavior in males, our results are relevant since they show that a gap exists between females and males access to specialist assessment in ED and call for gender‐specific strategies to warrant early mental health assessment in ED. Also, it is possible that ED staff might differently address psychiatric symptoms in females versus males, offering a more thorough assessment process to females. Females could also more frequently access to ED because of higher rates of anxious symptoms. One further reason behind higher sedative treatment in males is that males that access to ED more frequently have poor medication adherence which might be associated with more disturbed behavior (Juhás & Agyapong, 2016).

In addition, despite such severe clinical presentations, a psychiatric consultation is requested only in a portion of patients (2.41%) who are treated with anxiolytic treatment (4.66%). Several reasons could explain why medical doctors working in ED do not always require a psychiatric consultation when a subject presents psychiatric symptoms. First, a detailed diagnostic process is not a priority in ED; excluding emerging life‐threatening risks is the main aim of ED instead. Such a priority hierarchy might restrict the clinical focus on managing emerging anxiety symptoms, rather than in starting a thorough diagnostic process. Second, frantic, chaotic, and unpredictable work pace in ED may push the symptoms threshold to request specialist (i.e., psychiatric) consultation well above a typical outpatient service setting. Third, waiting time reduction is a priority of MDs working in ED, both to minimize service users' distress, and to minimize the risk of undertreating clinical pictures initially presenting as mild/moderate which may eventually worsen (i.e., green codes evolving to yellow or red codes) and to minimize service users' distress. Hence, quick pragmatic treatment approaches might be preferred over time‐consuming interviews and diagnostic processes. Fourth, on the other hand, some patients who may have accessed to ED because of severe self‐harm or severe trauma and who may be in need of psychopharmacologic treatment because of anxiety or agitation, can be transferred to intensive care unit without any psychiatric consultation, which in turn is then delivered subsequently after life threatening risk is excluded. Missing a specialist evaluation when needing psychopharmacological treatment may contribute to the lack of recognition of anxiety, depressive, or subthreshold psychotic symptoms which are largely prevalent in subjects with At Risk Mental State (Rutigliano et al., 2016).

Moreover, data from the real‐world large sample included in the present study show that an increasing number of adolescents and young subjects have been referring to ED from 2007 to 2017, suggesting that a *jatus* exists in mental health‐care of young subjects, and in adolescents in particular given the organization of adult mental health services in Italy which only assess and treat subjects older than 18 years old. Such increase in adolescents and young subjects referral to ED in a European country (Italy) is in line with what has been recently reported from a nationally representative data set in the United States (Burstein et al., 2019), and from other countries (Ferrer et al., 2021). Importantly, given that around half of the subjects included in this study did not have a severe/moderate clinical presentation, they could have accessed to alternative services without barriers to access, and without any age threshold. Unfortunately, such services were not available in the area where the study was set between 2007 and 2017.

Several approaches have been proposed to optimize treatment of patients affected by mental illness in ED with acute presentations. In the UK, the Psychiatric Decisions Unit (PDU), an acute mental health unit which provides an additional non‐bedded facility for up to eight patients after first triage assessment, offers a dedicated setting to evaluate psychiatric symptoms, with preliminary evidence of a decrease in inpatients admissions (Trethewey et al., 2019). However, such solutions require further resource allocation which may not be immediately available across different clinical settings and health organizations.

On the other hand, beyond treatment, improving psychiatric assessment in ED in adolescents remains crucial for preventive psychiatry and early intervention (Hartmann et al., 2019; McGorry et al., 2018), and in particular given the ineffectiveness of prevention services to detect subjects who later develop psychosis (van Os & Guloksuz, 2017). ED could serve as an additional setting to screen for early symptoms of full blown psychiatric disorders, or for subthreshold symptoms. Compared with the creation of ad‐hoc units (which of course also have the role of treating patients and reducing psychiatric admissions, going beyond an improvement in psychiatric assessment), cost‐effective (Ising et al., 2015, 2017) alternatives are there to screen adolescents and young adults accessing to ED. For instance, quick self‐administered screening questionnaire for risk of psychosis, or other severe mental illness might be considered (Addington et al., 2015; Kline et al., 2015). Among those with a quick and self‐report structure, which are more likely implementable in the context of ED, Prodromal Questinonaire‐16 (PQ‐16) (de Jong et al., 2018; Lorenzo et al., 2018; Pelizza et al., 2019) and PRIME‐Revised (Kobayashi et al., 2008) are two options that can take as short time as 3 min, and that may improve psychiatric assessment in the context of ED without additional workload nor interference in ordinary or extra‐ordinary activities of ED team. Beyond psychosis, brief instruments to assess the risk of bipolar disorder have also been recently validated (Van Meter et al., 2019).

Among help‐seeking subjects (Fusar‐Poli, Díaz‐Caneja, et al., 2016; Fusar‐Poli, Schultze‐Lutter, et al., 2016; Rutigliano et al., 2016) who refer to ED with (subthreshold) psychiatric symptoms that go or do not go under a psychiatric consultation but have not already developed SMI, a portion could also have a drop in psycho‐social functioning, and could eventually meet criteria for being at risk for mental illnesses. Specifically, rather than focusing on subjects at risk for psychosis only, ED might work to detect symptoms suggesting a risk of different disorders. Recently a shift from “at risk for psychosis” state towards an “at risk for mental illness” state including a wider range of clinical pictures have been proposed, and such a state of risk for a wider set of mental disorders has been names “Clinical High At Risk Mental State” (CHARMS) (McGorry et al., 2018). In support of the role of ED as a potential setting for early detection of CHARMS, evidence has shown that subjects who are referred to prevention services by ED have an increased risk of psychosis compared with self‐referring subjects, for instance (Fusar‐Poli, Schultze‐Lutter, et al., 2016). If ED referral is a risk‐enrichment factor for developing severe mental illness, a proper psychiatric assessment should be warranted in help‐seeking subjects in ED. This is important in particular given epidemiologic evidence showing that around 20% of adolescents accessing to ED for psychiatric symptoms presents with self‐harm behavior, which is a transdiagnostic symptom that deserves a thorough clinical examination and diagnostic assessment (Ferrer et al., 2021). Not warranting proper psychiatric assessment or at least referral to other services in ED can lead to missing early detection and intervention for a considerable number of adolescents with emerging psychiatric symptoms.

The present work has several points of strength. First, data are based on a large sample size. Second, data are representative of a time lapse as long as 10 years, well representing “real world” going beyond short time trends. Third, measurement of outcomes was not prone to bias since all medical evaluations and prescriptions were electronically recorded. Fourth, it assesses predictors of psychiatric consultations and psychopharmacological treatment in young subjects aged 15 to 24 years old referring to ED.

The present study also has several limitations. First, the study is based on real‐world data, which have both advantages but also important limitations (Corrigan‐Curay et al., 2018). Second, the role of more finely grained definitions of main reasons for referral to ED (i.e., abdominal pain, headache, fever, seizures, diarrhea, allergic reactions, etc…) in predicting psychiatric consultation and psychopharmacological treatment has not been considered. Third, several potential confounding factors such as substance abuse, psychomotor agitation, family history of psychiatric disorder, among others had not been collected in this cohort study. Fourth, an investigation of factors associated with a more fine‐grained definition of psychiatric diagnosis after psychiatric consultation, treatment, or admission within ED is not provided. Fifth, a longer follow‐up evaluation on eventual psychiatric disorder persistence or onset after first ED access is missing. Sixth, we did not investigate the reason for the increased rates of psychiatric consultation or treatment over time.

In conclusion, we show that an increasing number of young patients aged between 15 and 24 years old refer to ED with psychiatric symptoms, that females more likely show help‐seeking behavior and undergo a psychiatric consultation, that they have severe clinical pictures requiring urgent medical evaluation and intense resource utilization, and that only a portion of patients presenting psychiatric symptoms in ED is evaluated by a psychiatrist. Future studies should evaluate what the specific psychiatric outcome of young subjects referring to ED with psychiatric symptoms is, what finer grained factors are associated with need of psychiatric evaluation or treatment (i.e., pain or other somatic symptoms), what specific strategies could work to fill the gap in early mental health assessment between males and females, and ultimately whether ED may work as a setting for early detection of subjects with CHARMS who later develop SMI.

## CONFLICT OF INTEREST

Dr. Solmi received honoraria from Lundbeck and has been a consultant for Angelini on the advisory board. He is also a Joint Editor for JCPP *Advances*. Other authors have no conflict of interest to declare. [Corrections made on 22 June 2022, after first online publication: This Conflict of Interest statement has been updated in this version.]

## AUTHORS CONTRIBUTION

Carla Cremonese, Foscarina Della Rocca, Marco Solmi designed the study. Marco Solmi, Umberto Granziol conducted analyses. All authors substantially contributed to the manuscript, approved and take responsiblity for the work.

## ETHICAL STATEMENT

The present study adhered to Helsinki Declaration. Subjects consented to use data in aggregated anonymous form.

## Supporting information

Supplementary Material

## Data Availability

Data are available upon request with a pre‐specified research question.
