# Supplementary material for: Increasing referrals to emergency department for psychiatric consultation and treatment among 50,056 adolescents and young adults: Predictors and implications
Source: JCPP Adv. 2021 Jun 26;1(2):e12016. doi: 10.1111/jcv2.12016 (PMC10242815; doi:10.1111/jcv2.12016)
Supplement: Supplementary file 1 — Supplementary Material [file JCV2-1-e12016-s001.docx]

Online Supporting Information for: *Increasing referrals to Emergency Department for psychiatric consultation and treatment among 50,056 adolescents and young adults: predictors and implications* – by Solmi et al.

**Appendix S1.**

R CODE

library(lme4)

mydata<-read.csv(file.choose(),sep = ";",header = T)

#### Outcome: Psychiatric Consultation

Consultation<-mydata

Consultation$Referral_modality<-factor(Consultation$Referral_modality = c("Autonomy "," Other "," Ambulance without doctor "," Ambulance with doctor "))

Consultation$Triage_Code<-factor(Consultation$Triage_Code,levels = c("White "," Green "," Yellow"," Red "))

Model_consultation<-glmer(Consultation~Gender+Age+Triage_Code+ Referral_modality +(1|Subject),family = binomial(link = "logit"),data=Consultation, control = glmerControl(optimizer = "bobyqa",optCtrl = list(maxfun=2e6)))

r.squaredGLMM(Model_consultation)

Coefficients_Model_consultation<-as.data.frame(coef(summary(Model_consultation)))

Conf_Int<-confint(Model_consultation,parm="beta_",method="Wald")

Odd_ratios <- cbind(OR=fixef(Model_consultation), Conf_Int)

rtab <- round(exp(Odd_ratios),3)

Results_1<-cbind(rtab,round(Coefficients_Model_consultation $`Pr(>|z|)`,7))

Results_1

#### Outcome: Anxiolytic Treatment

Anxiolytic<-mydata

Anxiolytic $Referral_modality<-factor(Anxyiolitic $modarr,levels = c("Autonomy "," Other "," Ambulance with doctor "," Ambulance without doctor "))

Anxiolytic $Triage_Code<-factor(Anxyiolitic $Triage_Code,levels = c("White "," Green "," Yellow"," Red "))

Model_ Anxiolytic <-glmer(Anxiolytic ~Gender+Age+Triage_Code + Referral_modality +(1|Subjsect),family = binomial(link = "logit"),data= Anxiolytic, control = glmerControl(optimizer = "bobyqa",optCtrl = list(maxfun=2e6)))

r.squaredGLMM(Model_ Anxiolytic)

Coefficients_Model_ Anxiolytic <-as.data.frame(coef(summary(Model_ Anxiolytic)))

Conf_Int<-confint(Model_ Anxiolytic,parm="beta_",method="Wald")

Odd_ratios <- cbind(OR=fixef(Model_ Anxiolytic), Conf_Int)

rtab <- round(exp(Odd_ratios),3)

Results_2<-cbind(rtab,round(Coefficients_Model_ Anxiolytic $`Pr(>|z|)`,7))

Results_2

#### Outcome: Sedative Treatment

Sedative<-mydata

Sedative $Referral_modality<-factor(Sedative $modarr,levels = c("Autonomy "," Other "," Ambulance without doctor "," Ambulance with doctor "))

Sedative $Triage_Code<-factor(Sedative $Triage_Code,levels = c("White "," Green "," Yellow"," Red "))

Model_ Sedative <-glmer(Sedative ~Gender+Age+Triage_Code+Referral_modality +(1|Subjsect),family = binomial(link = "logit"),data= Sedative, control = glmerControl(optimizer = "bobyqa",optCtrl = list(maxfun=2e6)))

r.squaredGLMM(Model_ Sedative)

Coefficients_Model_ Sedative <-as.data.frame(coef(summary(Model_ Sedative)))

Conf_Int<-confint(Model_ Sedative,parm="beta_",method="Wald")

Odd_ratios <- cbind(OR=fixef(Model_ Sedative), Conf_Int)

rtab <- round(exp(Odd_ratios),3)

Results_3<-cbind(rtab,round(Coefficients_Model_ Sedative $`Pr(>|z|)`,7))

Results_3

#### Outcome: Psychiatric admission

Admission<-mydata

Admission $Referral_modality<-factor(Admission $modarr,levels = c("Autonomy "," Other "," Ambulance without doctor "," Ambulance with doctor "))

Admission $Triage_Code<-factor(Admission $Triage_Code,levels = c("White "," Green "," Yellow"," Red "))

Model_ Admission <-glmer(Admission ~Gender+Age+Triage_Code + Referral_modality +(1|Subjsect),family = binomial(link = "logit"),data= Admission, control = glmerControl(optimizer = "bobyqa",optCtrl = list(maxfun=2e6)))

r.squaredGLMM(Model_ Admission)

Coefficients_Model_ Admission <-as.data.frame(coef(summary(Model_ Admission)))

Conf_Int<-confint(Model_ Admission,parm="beta_",method="Wald")

Odd_ratios <- cbind(OR=fixef(Model_ Admission), Conf_Int)

rtab <- round(exp(Odd_ratios),3)

Results_4<-cbind(rtab,round(Coefficients_Model_ Admission $`Pr(>|z|)`,7))

Results_4

library(tfplot)

library(tseries)

library(pastecs)

library(ggplot2)

library(gridExtra)

#### Time series Analyses

# Psychiatric consultation

Con_psi<-ts(con,start = c(2007,1),end = c(2016,1))

Annual_change_con_psy <- annualizedGrowth(Con_psi)

trend.test(Con_psi)

trend.test(Con_psi,R=999)

# Anxyolitic Treatment

Anx_treat<-ts(at,start = c(2007,1),end = c(2016,1))

Annual_change_ Anx_treat <-annualizedGrowth(Anx_treat)

trend.test(Anx_treat)

trend.test(anx Anx_treat R=999)

# Sedatve Treatment

Sed_treat<-ts(sdt, start = c(2007,1),end = c(2016,1))

Annual_change _ Sed_treat <-annualizedGrowth(Sed_treat)

trend.test(Sed_treat)

trend.test(Sed_treat, R=999)

# Psychiatric admission

Psyc_adm<-ts(spc, start = c(2007,1),end = c(2016,1))

Annual_change _ Psyc_adm <-annualizedGrowth(Psyc_adm)

trend.test(Psyc_adm)

trend.test(Psyc_adm,R=999)
